# Methicillin-Resistant *Staphylococcus aureus*: The Shifting Landscape in the United Arab Emirates

**DOI:** 10.3390/antibiotics14010024

**Published:** 2025-01-02

**Authors:** Syrine Boucherabine, Rania Nassar, Lobna Mohamed, Maya Habous, Anju Nabi, Riyaz Amirali Husain, Mubarak Alfaresi, Seema Oommen, Hamda Hassan Khansaheb, Mouza Al Sharhan, Handan Celiloglu, Mubarak Hussain Raja, Eman Abdelkarim, Nishi Ali, Salman Tausif, Victory Olowoyeye, Nelson Cruz Soares, Mahmood Hachim, Danesh Moradigaravand, Dean Everett, Elke Mueller, Stefan Monecke, Ralf Ehricht, Abiola Senok

**Affiliations:** 1 College of Medicine, Mohammed Bin Rashid University of Medicine and Health Sciences, Dubai P.O. Box 505055, United Arab Emirates; rania.nassar@dubaihealth.ae (R.N.); lobna.mohamed@dubaihealth.ae (L.M.); hhkhansaheb@dubaihealth.ae (H.H.K.); victory.olowoyeye@residents.mbru.ac.ae (V.O.); mahmood.almashhadani@dubaihealth.ae (M.H.); abiola.senok@dubaihealth.ae (A.S.); 2 Microbiology & Infection Control Unit, Pathology Department, Rashid Hospital, Dubai P.O. Box 4545, United Arab Emirates; mmhabou@dubaihealth.ae (M.H.); mrzeeshan@dubaihealth.ae (M.H.R.); 3 Microbiology Department, Dubai Hospital, Dubai P.O. Box 7272, United Arab Emirates; anabi@dubaihealth.ae (A.N.); rahusain@dubaihealth.ae (R.A.H.); maalsharhan@dubaihealth.ae (M.A.S.); 4 Department of Microbiology, National Reference Laboratory, Abu Dhabi P.O. Box 92323, United Arab Emirates; mubarak.alfarsi@msc.mil.ae; 5 Department of Microbiology, Pathology and Laboratory Medicine Institute, Cleveland Clinic, Abu Dhabi P.O. Box 112412, United Arab Emirates; 6 Laboratory Department, Burjeel Medical City/CoLAB, Abu Dhabi 92510, United Arab Emirates; drseema@burjeelmedicalcity.com; 7 Microbiology Department, Mediclinic City Hospital, Dubai Healthcare City, Dubai P.O. Box 31500, United Arab Emirates; handanyol@hotmail.com (H.C.); nishi.ali@mediclinic.ae (N.A.); salman.tausif@mediclinic.ae (S.T.); 8 Dubai Academic Health Corporation, Dubai P.O. Box 505055, United Arab Emirates; emanfathi369@gmail.com; 9 Center for Applied and Translational Genomics (CATG), Mohammed Bin Rashid University of Medicine and Health Sciences, Dubai Health, Dubai P.O. Box 505055, United Arab Emirates; nelson.soares@dubaihealth.ae; 10 Laboratory of Proteomics, Department of Human Genetics, National Institute of Health Doutor Ricardo Jorne (INSA), 4055 Lisbon, Portugal; 11 Center for Toxicogenomics and Human Health (toxOMICS), NOVA School/Faculdade de Lisboa, 1099-085 Lisboa, Portugal; 12 Laboratory of Infectious Disease Epidemiology, Biological and Environmental Science and Engineering (BESE) Division, King Abdullah University of Science and Technology (KAUST), Thuwal P.O. BOX 4700, Saudi Arabia; danesh.moradigaravand@kaust.edu.sa; 13 Department of Public Health and Epidemiology, College of Medicine and Health Sciences, Khalifa University, Abu Dhabi P.O. Box 127788, United Arab Emirates; dean.everett@ku.ac.ae; 14 Biotechnology Center, Khalifa University, Abu Dhabi P.O. Box 127788, United Arab Emirates; 15 Infection Research Unit, Khalifa University, Abu Dhabi P.O. Box 127788, United Arab Emirates; 16 Leibniz Institute of Photonic Technology (IPHT), Leibniz Center for Photonics in Infection Research (LPI), 07745 Jena, Germany; elke.mueller@leibniz-ipht.de (E.M.); stefan.monecke@leibniz-ipht.de (S.M.); ralf.ehricht@leibniz-ipht.de (R.E.); 17 InfectoGnostics Research Campus Jena, 07743 Jena, Germany; 18 Institute of Physical Chemistry, Friedrich Schiller University Jena, 07737 Jena, Germany; 19 School of Dentistry, Cardiff University, Cardiff CF14 4XY, UK

**Keywords:** MRSA, genotyping, microarray, antimicrobial resistance, *Staphylococcus aureus*

## Abstract

Background: Methicillin-resistant *Staphylococcus aureus* (MRSA) is a significant burden globally, particularly in the Arabian Gulf region. The United Arab Emirates (UAE) has experienced rising MRSA prevalence, with increasing diversity in the clonal complexes (CCs) identified. The COVID-19 pandemic, with its increased hospitalization rates and antibiotic use, may have further influenced MRSA’s genetic evolution and epidemiology in the country. Methods: To investigate this influence, genomic profiling of 310 MRSA clinical isolates collected between February and November 2022 was performed using a DNA microarray-based assay. Results: Isolates were assigned to 22 clonal complexes and 72 distinct strain assignments. The predominant clonal complexes were CC5, CC6, CC361, CC22, CC1, and CC8. Community-acquired MRSA lineages were dominant, with only one healthcare-associated MRSA lineage isolate identified. Upward trends of CC1153 were observed along with rare CCs, such as CC121-MRSA and CC7-MRSA, with the latter being reported for the first time in the Arabian Gulf region. The presence of pandemic strains USA300 CC8-MRSA-[IVa + ACME1] and CC8-MRSA-IV strains were also observed, including variants lacking Panton–Valentine leukocidin (*pvl*) genes and missing *tst1* or enterotoxin genes. The PVL-negative CC772-MRSA-V/VT was identified, representing its first report in the UAE. A novel variant, CC361-MRSA-IV (*tst1*+/PVL+), was identified. *Pvl* genes were observed in 36% of the isolates, primarily from skin and soft tissue infections, while *fusC* (SCC-borne fusidic acid resistance) was identified in 13% of the isolates. Conclusions: The findings highlight the ongoing evolution of MRSA in the UAE, with the persistence and emergence of diverse and rare clonal complexes, driving the need for continuous genomic surveillance.

## 1. Introduction

Methicillin-resistant *Staphylococcus aureus* (MRSA) remains one of the major causes of community-acquired and healthcare-associated infections, leading to increased morbidity, mortality, and healthcare costs [1]. MRSA first emerged in England in the 1960s and has since become a global public health concern, with it being listed as a priority antimicrobial resistance pathogen by the World Health Organization. MRSA strains exhibit considerable genetic diversity, reflected in the wide array of clonal complexes and strain assignments, with many strains harboring significant virulence genes as well as genes that confer multidrug resistance. There is an ongoing shift from hospital- to community-associated (CA) MRSA lineages as etiological agents of nosocomial infections in most healthcare settings [2,3].

MRSA contributes significantly to the burden of antimicrobial resistance (AMR) in the countries of the Arabian Gulf region, with prevalence rates ranging from 15 to 55% depending on the study setting [2,4,5,6,7,8]. In the United Arab Emirates (UAE), longitudinal twelve-year national AMR surveillance data have shown that the prevalence of MRSA infections increased from 21.9% in 2010 to 33.5% in 2021 [9]. MRSA infections have also contributed to the healthcare burden by increasing the length of hospitalization and the rate of intensive care unit (ICU) admissions [9]. This is particularly concerning considering the robust infection prevention and control protocols implemented in the healthcare system. Data from molecular characterization of MRSA in the countries of the Arabian Gulf region including the UAE indicate that the majority of healthcare-associated infections are caused by CA-MRSA lineages with wide clonal diversity [4]. There is also widespread distribution of MRSA isolates harboring the SCC*mec* + SCC*fus* composite elements which provide a selective advantage in both hospital and community settings [2,10,11]. Furthermore, the high occurrence of strains harboring virulence genes such as Panton–Valentine leukocidin (*pvl*) genes and those with a multidrug-resistant genetic profile is of significant concern [12]. The UAE’s position as a global hub with a large expatriate population may contribute to the introduction and circulation of diverse MRSA clones into the country.

Considering the heightened rates of hospitalization and antimicrobial consumption globally during the COVID-19 pandemic, a worsening of antimicrobial resistance trends has been predicted [13,14,15]. The need for ongoing AMR surveillance incorporating genomic profiling has also been highlighted. Currently, there is insufficient data on the molecular characterization of MRSA in the UAE. Isolates obtained prior to the pandemic showed wide clonal diversity, with the emergence of previously unreported novel and variant strains [2]. In addition, genomic surveillance, which could shed light on the molecular landscape of MRSA in the UAE to identify drivers of dissemination and inform the development of targeted preventive strategies, is largely lacking. Our review of the literature did not identify any report on the genomic profile of MRSA isolates circulating in the UAE in the pandemic/post-pandemic period. Therefore, to address this gap in the literature, this study was undertaken to determine the genomic profiling of MRSA isolates circulating in the UAE during the pandemic/post-pandemic period with a view to elucidating genetic diversity, virulence factors, and resistance mechanisms.

## 2. Results

A total of 310 MRSA isolates predominantly from skin and soft tissue infections (n = 121), respiratory tract specimens (n = 37), blood culture (n = 23), eye/ear discharge (n = 29), and abscesses/pus (n = 55) were analyzed, as well as 36 isolates from other, less common types of clinical specimens and 9 for which no details were provided by the submitting wards. Table 1 shows the demographic profile of the patients from whom these isolates were obtained. The isolates were assigned to 22 clonal complexes (CCs) and 72 distinct strains. Detailed strain assignments for each clonal complex along with the SCC*mec* types are shown in Table 2. Identified SCCmec types can be found in Supplementary Materials Table S1. The predominant CCs identified were CC5 (n = 49), CC6 (n = 45), CC361 (n = 29), CC22 (n = 27), CC1 and CC8 (n = 23 each), and CC30 (n = 21).

The SCC-borne fusidic acid resistance gene (*fusC*) was detected in 14% (n/N = 44/310) of the isolates. Other resistance genes detected include the erythromycin/clindamycin resistance gene e*rmC* (n/N = 28/310; 9%), the gene encoding a bifunctional aminoglycoside-modifying enzyme (*aacA-aphD*) (n/N = 42/310; 13.5%), and the mupirocin resistance gene *mupA* (n/N = 28/310;9%). None of the strains carried vancomycin resistance genes (*vanA* and *vanB*) and there was concordance between the genotypic and phenotypic resistance profiles of the isolates. Table 3 shows the distribution of the antibiotic-resistance genes identified. A high carriage of virulence genes was also observed, with the presence of the Panton–Valentine leukocidin *(pvl*) genes detected in 38% (n/N = 120/310) of isolates, with 70% (n/N = 85/120) of them being from skin and soft tissue infections and three cases being from patients with severe pneumonia. The toxic shock syndrome gene (*tst-1*) was present in 12.2% (n/N = 38/310) of the isolates. Both *pvl* and *tst-1* genes were present simultaneously in 19 isolates. The prevalence rates of the virulence genes are shown in Table 4.

## 3. Discussion

Globally, MRSA genomic data have shown continuous evolution in its epidemiology. This trend is also evident in the Arabian Gulf region, with reports from the UAE, Saudi Arabia, Kuwait, and Qatar before the onset of the COVID-19 pandemic documenting changes in MRSA’s evolution and epidemiology, including wide clonal diversity as well as the emergence of novel, rare, and variant MRSA strains [2,6,8,16,17]. This unique MRSA epidemiology has often been associated with the dynamic population movement seen across the countries of the Arabian Gulf region, most of which are global hubs for tourism and business as well as home to large expatriate communities.

Our findings demonstrate that there remains an extensive MRSA repertoire circulating in the UAE with continued predominance of CC1, CC5, CC6, CC8, CC22, CC30, and CC361. These findings are in accordance with previously reported work from the UAE and across the region, including a recent report from Kuwait which documented isolates circulating in the early pandemic period [5]. Our results also show that presumably “community-associated” lineages, i.e., those with SCC*mec* types IV and V, continue to drive nosocomial infections in healthcare settings in the UAE, and indeed, only one isolate belonging to an HA-MRSA lineage (CC239) was identified in this study. The previously predominant CCs in the UAE, which include CC80, CC97, CC239, and CC779 [2], were detected in smaller numbers in this study. This is in contrast to the observations for C1153. This lineage was described as an emerging CC in previous reports, and findings from this study also indicate an upward trend [2,18]. This suggests that CC1153 has now become a common and well-established MRSA CC in our setting [19]. Pandemic strains such as a variant of USA300 CC8-MRSA-[IVa + ACME1] lacking *pvl* genes and CC8-MRSA-IV missing *tst1* or enterotoxin genes were also identified. One CC121-MRSA-V/VT (PVL+) and two CC121-MRSA-[V/VT + *fusC*] (PVL+) were detected. This is remarkable as CC121 MRSA is rare (while CC121 MSSA is globally abundant) and it suggests the persistence of CC121 MRSA in the UAE, which is in accordance with reports from Kuwait [2,5]. In addition, CC7 MRSA, which was first identified in Saxony and Australia, represents another rare MRSA CC [20] detected in this study. The finding of three CC7 MRSA isolates with two being the CC7-MRSA-V/VT strain (resembling WA-MRSA-131) [21] represents the first report from the UAE and the Arabian Gulf region. The CC7-MRSA-V/VT strain has been previously linked with livestock as it has been reported in two pig farms in Norway [22], and it was identified as a colonizer in students in Nigeria [23].

The predominant CC in this study was CC5, which, globally, is a common clonal complex [20]. The majority of CC5 strains identified in this study were CC5-MRSA-[V/VT + *fusC*] (*sed/j/r−).* Although similar strains have been previously reported in Saudi Arabia, Australia, and Ireland [8,24], this represents the first report from the UAE. In addition, we also identified CC5-MRSA-IV (*se/d/j−*, *tst−*, *pvl−*, *edinA−*) “Paediatric clone/WA MRSA-74” which had not been previously reported in the UAE. These findings indicate an expansion of the repertoire of CC5 MRSA and suggest ongoing shifts in the predominant strain circulating in the country. The continued prevalence of previously reported CC5-MRSA-[V/VT + *fusC*] (PVL+), the “Maltese clone” CC5-MRSA-[IV + *fusC* + *ccrAB*] [25], and the ST5/ST225-MRSA-II, Rhine-Hesse EMRSA/New York-Japan Clone [26] indicates that CC5 MRSA is now endemic within the healthcare system in the UAE. The second most common CC was CC6, with the majority having SCC*mec* IV and thus resembling Australian WA MRSA-51 [27]. Although the first report of WA MRSA-51 in the Arabian Gulf region was in Abu Dhabi [28], other studies have identified this strain as one of the main contributors to MRSA epidemiology in the UAE and Saudi Arabia [2,29]. CC1 MRSA, which is known to be inclusive of multiple CA-MRSA strains, was also very prevalent. The most commonly identified CC1 strains in this study were CC1-MRSA-[V/VT + *fusC* + *ccrAB1*] and CC1-MRSA-[V/VT + *fusC* + *ccrAB1*] (PVL+), which have been reported before in the UAE, Kuwait, and Egypt [2,16,30]. Another strain from this clonal complex was CC1-MRSA-PseudoSCC*mec* [classC + *fus* + *ccrAB1*], which harbors a pseudoSCC*mec*, an SCC*mec* without the recombinase (*ccr*) genes [31] (whereas *ccrAB1* genes are likely associated with *fusC*). A similar strain has been identified in Kuwait but with a class B pseudoSCC*mec* [5].

MRSA CC152 was initially described as CA MRSA in Europe, characterized by the carriage of the SCC*mec* type V and *pvl* genes [20]. However, reports from Africa have shown its dissemination along with its presence in four countries [32], and it was later documented in recent reports from Kuwait and the UAE [2,5]. CC152 methicillin-susceptible/*mecA*-negative *S. aureus* strains have been shown to be prevalent across Africa [32], and it is likely that CC152 MRSA also emerged there with subsequent spread to other parts of the world.

Previously, CC361 MRSA in the UAE was considered rare as a single strain was isolated in 2009 [20,28]. However, a recent report from the UAE showed that CC361 represented 5% of the total collection of isolates, indicating that this lineage established itself in our setting [2]. Findings from this study indicated the continued expansion of this CC in our setting as 9.4% (n/N = 29/310) of isolates were CC361. In addition, our observations demonstrate that this expansion is being driven predominantly by CC361-MRSA-[V/VT + *fusC*] followed by CC361-MRSA-V/VT and WA MRSA-70/110 strains. Moreover, a novel strain CC361-MRSA-IV (*tst1*+/PVL+) was identified in this study. This represents the first report of CC361-MRSA-IV with *pvl* genes, indicative of an ongoing evolution of this clonal complex in our setting.

The CC398-MRSA isolate identified in this study belonged to the PVL-positive human-associated variant (as opposed to the PVL-negative European livestock-associated CC398). As previously reported, this lineage is believed to have originated from Southeast Asia, with infections by this isolate being reported in China [33] and Vietnam [34] as well as Southeast Asian emigrants in Europe [35]. This is the second observation of the human variant of the CC398 in the UAE [2], and there are no published reports indicating its identification in other countries in the Arabian Gulf region. Genomic sequencing to better understand how this CC398 variant differs from the livestock-associated lineage is needed.

CC772-MRSA-V, also known as the Bengal Bay Clone, which first emerged on the Indian subcontinent, has been previously reported in multiple places such as Australia, several European countries, Hong Kong, Malaysia, and the UAE [2,20]. The majority of CC772 isolates identified in this study were PVL positive, which is in accordance with previous reports from the UAE and the region [2,16,29]. Interestingly, the PVL-negative CC772-MRSA-V/VT strain, which has only been previously reported from Nepal [36], was detected for the first time in the UAE. However, although there is a strong epidemiological link between Nepal and the UAE, this strain was identified in a patient from Africa. This finding highlights the importance of genomic surveillance work in a global hub like the UAE as it can provide important information about MRSA strains in regions where we have limited data available.

The recently described species *S. argenteus* is genetically close to *S. aureus* and other coagulase-positive *Staphylococcus* species [37]. The *S. argenteus* CC2250 identified in this study has been reported in multiple parts of the world, including Australia (“WA MRSA-114”) and our region [38].

The MRSA isolates showed an extensive repertoire of virulence factors, with 36% being positive for *pvl* genes, while the *tst1* gene was found in 12.3%. Even though this is lower than what had previously been reported [2], the previous study was over a period of two years and had a higher number of isolates. The high occurrence of MRSA isolates with carriage of *pvl* genes in our region is a concern, especially as most are associated with skin and soft tissue infections. We advocate for the introduction of rapid diagnostic tests for PVL detection in MRSA to guide therapeutic choices [39]. The prevalence of the *tst1* gene was majorly observed in isolates belonging to CC22, which is in concordance with previous reports of the region as the strain is widely distributed in the Middle East [29,40]. Other virulence factors coding for biofilm formation, epidermal cell differentiation inhibitors (*edin*), and various enterotoxins were also present in our collection. The *edin* genes set has been widely reported to be associated with CA-MRSA lineages in Europe and Tunisia in concordance with *pvl* carriage, with most isolates being associated with diabetic foot ulcers, which could explain the high carriage in our isolates [41,42]. We also report the presence of the *SCC*-borne fusidic acid resistance (*fusC*) gene in 14% of the isolates, which is in concordance with previous reports from the country and region [2,6,16,18,43,44]. SCC*mec*-linked fusidic acid resistance has been widely reported in the Arabian Gulf region possibly due to the extensive use of this topical antibiotic for SSTIs, and indeed, such use has been linked with increased resistance in New Zealand [11].

During the COVID-19 pandemic, the dynamics of population movement were altered as lockdowns were implemented across international borders as well as between cities and regions within many countries. In addition to increased hospitalization rates and antibiotic use during the pandemic, these changes in patterns of population movement could contribute to the evolution of antimicrobial-resistant pathogens [13]. MRSA contributed to the burden of AMR infections in the UAE during the COVID-19 pandemic [45]. Our findings provide insights into the genomic profile of MRSA isolates in our setting during the latter part of the pandemic period, with the lower detection of rare and previously unreported strains as well as novel variants compared to previous reports from the UAE [2]. We hypothesize that travel restrictions and social distancing during the pandemic might have played a role in limiting the introduction of new strains and the de novo emergence of variants.

A limitation of our study was the lack of additional patient information such as travel history and prior antibiotic use. Hence, correlations between genomic characterization and clinical parameters could not be made. For future work, it will be useful to obtain such additional information, which might provide insights to corroborate genomic data with clinical parameters.

In conclusion, our study shows a slight shift in MRSA distribution patterns in the United Arab Emirates, with a high degree of diversity. The high occurrence of community-associated MRSA lineages continues to drive MRSA evolution in our healthcare settings. The presence of strains that carry composite SCC*mec* and SCC*fusC* cassettes warrants attention due to their ability to flourish in community and healthcare settings. However, in the post-pandemic era and lifting of all travel restrictions, genomic surveillance and infection control practices are recommended to curb renewed dissemination of MRSA strains. We also recommend continued health education and nasal screening for MRSA as components of infection prevention.

## 4. Materials and Methods

### 4.1. Specimen Collection and Bacterial Strains

MRSA isolates included in the study were those identified between February and November 2022 at five diagnostic laboratories of secondary and tertiary care facilities in the UAE. These included two large private hospital networks and three governmental hospitals across three Emirates (Dubai, Abu Dhabi, and Umm Al Quwain). MRSA isolates associated with clinical infections were obtained, and only one isolate per patient was included in the study. There was no exclusion based on patient age. MRSA isolates obtained from screening specimens were excluded from the study. Bacterial identification, confirmation of MRSA, and phenotypic antibiotic resistance testing were performed by the submitting laboratories with the automated VITEK 2 system (bioMérieux, Marcy-l’Étoile, France) in accordance with manufacturer-provided protocols and Clinical and Laboratory Standards Institute guidelines [46]. Antibiotic susceptibility testing (AST) using VITEK^®^ 2 AST cards is based on the broth microdilution minimum inhibitory concentration technique. The isolates were stored in cryobead vials (MAST CRYOBANKTM, MAST, Bootle, UK) at −80 °C. As the isolates were those identified as part of routine diagnostic investigations, a waiver of informed consent was granted.

### 4.2. Molecular Characterization

**Figure 1:**
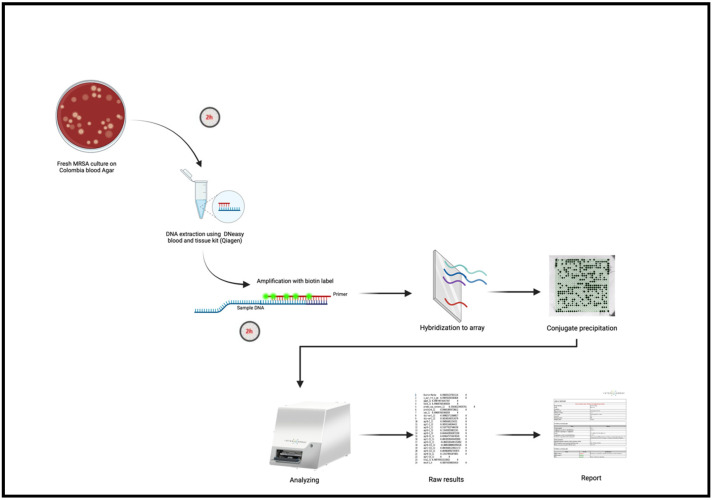
Flowchart of the steps of the DNA microarray assay (created using biorender.com).

The INTER-ARRAY Genotyping Kit *S. aureus* (Inter-Array GmbH, Bad Langensalza, Germany) was used for molecular characterization of the isolates as previously described and in accordance with manufacturer-provided protocols [20]. Briefly, isolates were recovered from frozen stocks and cultured on blood agar for 24 h. Colonies were obtained, and DNA extraction was carried out using a DNeasy blood and tissue kit (Qiagen, Hilden, Germany). Amplification and labeling of extracted DNA were carried out, with the resulting amplicons used for DNA hybridization, and array images were taken by a microarray reader (Inter-Array by Fzmb GmbH, Bad Langensalza, Germany). Figure 1 shows the flowchart of the DNA microarray-based analysis [47]. The array images taken by the reader were analyzed using a dedicated software and database (Inter-Array GmbH, Germany). Data analysis included assignment into clonal complex and strain as well as detection of carriage of target antibiotic resistance and virulence genes.

## Figures and Tables

**Table 1:** Demographic profile of patients.

Demographic Data	Total # of Patients (N = 310)
Age	
≥18	264 (85.2%)
<18	44 (14.2%)
Not reported	2 (0.6%)
Gender	
Female	114 (36.8%)
Male	194 (62.6%)
Not reported	2 (0.6%)
Hospitalization	
Inpatients	140 (45.2%)
Outpatients	143 (46.1%)
Not reported	27 (8.7%)
Nationality	
Non-Emiratis	218 (70.3%)
Emiratis	88 (28.4%)
Not reported	4 (1.3%)

**Table 2:** Distribution of MRSA clonal complexes and strain assignments.

Clonal Complex	Strain Assignment	Number of Strains
CC1 (n = 23) *	CC1-MRSA-[V/VT + *fusC*] (PVL+)	1
	CC1-MRSA-[IV + *fusC + ccrAB1*], WA MRSA-1/45	4
	CC1-MRSA-[V/VT + *fusC + ccrAB1*]	9
	CC1-MRSA-[V/VT + *fusC + ccrAB1*] (PVL+)	4
	CC1-MRSA-IV (*aphA3/sat*-positive)	2
	CC1-MRSA-IV, WA MRSA-1/57 (*aphA3*/*sat*-negative)	1
	CC1-MRSA-PseudoSCC*mec* [classC + *fus* + *ccrAB1*]	1
CC5 (n = 49)	CC5-MRSA-[V/VT + *fusC*] (*sed/j/r*−)	19
	CC5-MRSA-[VI + *fusC*]	2
	CC5-MRSA-IV *(sed/j/r−*, PVL+), “Sri Lanka Clone”	1
	CC5-MRSA-[V/VT + *fusC*] (PVL+)	6
	CC5-MRSA-IV (*tst*−, PVL−, *edinA*+)	4
	CC5-MRSA-IV (*tst*+, PVL−, *edinA*−)	1
	CC5-MRSA-V/VT (*sed*/*j*/*r*−, *edinA*+)	3
	CC5-MRSA-[V/VT + *fus*] (*sed/j/r−*)	5
	CC5-MRSA-V/VT (*sed/j/r−*), WA MRSA-81/85/86/123	2
	CC5-MRSA-[IV + *fusC + ccrAB*], Maltese Clone	3
	CC5-MRSA-IV (*se*/*d*/*j*−, *tst*−, PVL−, *edinA*−), “Paediatric clone/WA MRSA-74”	1
	ST5/ST225-MRSA-II (*sed/sej/ser*-positive variant), Rhine-Hesse EMRSA/New York-Japan Clone	2
CC6 (n = 45)	CC6-MRSA-IV, WA MRSA-51	44
	CC6-MRSA-IV (PVL+)	1
CC7 (n = 3)	CC7-MRSA-V/VT, WA-MRSA-131	2
CC8 (n = 18)	CC8-MRSA-[IV + ACME] (PVL+), USA300	4
	CC8-MRSA-IV (without *tst1* or enterotoxin genes)	2
	CC8-MRSA-V/VT	4
	CC8-MRSA-[IV + ACME], PVL-deletion mutant of USA300	2
	CC8-MRSA-[V/VT + *ccrAB4*]	1
	CC8-MRSA-[VI + *fusC*]	1
	CC8-MRSA-[IVF + *ccrAB4*]/-VI	1
	CC8-MRSA-[IV + ACME + *ccrAB*4]	1
	CC8-MRSA-[V/VT + *fusC*]	1
CC9 (n = 2)	CC9-ST834	2
CC15 (n = 10)	CC15-MRSA-[V/VT + fus]	10
CC22 (n = 27)	CC22-MRSA-IV (PVL+/*tst+*)	17
	CC22-MRSA-IV (*tst1+*), “Gaza Epidemic Strain”	2
	CC22-MRSA-IV (PVL+)	6
	CC22-MRSA-IV *(fnbB+*), related to UK-EMRSA-15/Barnim EMRSA	1
	CC22-MRSA-[VI + *fusC*]	1
CC30 (n = 21)	CC30-MRSA-IV (PVL+), Southwest Pacific Clone	11
	CC30-MRSA-[VI + *fusC*] (PVL+)	3
	CC30-MRSA-IV (PVL−/*tst1*−)	1
	CC30-MRSA-V/VT (PVL+), WA MRSA-124	4
	CC30-MRSA-[VI + *fusC*]	1
CC45 (n = 1)	CC45-MRSA-[IV + *fusC*]	1
CC72 (n = 2)	CC72-MRSA-V/VT, WA-MRSA-131	2
CC80 (n = 6)	CC80-MRSA-IV (PVL+)	3
	CC80-MRSA-IV	2
	CC80-MRSA-[pseudo SCC*mec* IV] (PVL+)	1
CC88 (n = 9)	CC88-MRSA-IV (PVL+)	5
	CC88-MRSA-IV	1
	CC88-MRSA-V/VT (PVL+), WA MRSA-117	3
CC96 (n = 2)	CC96-MRSA-IV	2
CC97 (n = 12) *	CC97-MRSA-[V/VT + *fusC*]	4
	CC97-MRSA-IV, WA MRSA-54/63	1
CC121 (n = 4)	CC121-MRSA-[V/VT + *fusC*] (PVL+)	2
	CC121-MRSA-V/VT (PVL+)	2
CC152 (n = 16)	CC152-MRSA-[V/VT + *fusC*] (PVL+)	16
CC239 (n = 1)	CC239-MRSA-[III + *ccrC*]	1
CC361 (n = 29)	CC361-MRSA-[V/VT + *fusC*]	20
	CC361-MRSA-IV, WA MRSA-29	1
	CC361-MRSA-V/VT, WA MRSA-70/110	7
	CC361-MRSA-IV (*tst1*+/PVL+) ***	1
CC398 (n = 4)	CC398-MRSA-V/VT (PVL+)	4
CC772 (n = 9)	CC772-MRSA-V/VT (PVL+), “Bengal Bay Clone”	8
	CC772-MRSA-V/VT (PVL-)	1
CC1153 (n = 15) ***	CC1153-MRSA-[V/VT + *fusC*] (PVL+)	12
	CC1153-MRSA-V/VT (PVL+)	1
	CC1153-MRSA-PseudoSCC*mec* [class B + *fus* + *ccrAB1*] (PVL+)	2
CC2250 *S. argenteus* (n = 2) ^#^	CC2250-MRSA-IV, WA MRSA-114	2

* No strain assignment for 7 isolates; *** novel variant strain; ^#^ phenotypically identified as MRSA but found to be S. argenteus CC2250 upon genotyping.

**Table 3:** Detection of antibiotic resistance genes.

Antibiotic Resistance Genes		# Positive (N = 310)	% Positive
*mecA*	Alternate penicillin-binding protein 2a	310	100.0
*merA*; *merB*	Mercury resistance operon	6	1.9
*blaZ*; *blaI*; *blaR*	Beta-lactamase operon	270	86.8
*ermA*	R-RNA adenine n-6-methyl-transferase, Erythromycin/clindamycin resistance	5	1.6
*ermB*	Erythromycin/clindamycin resistance	46	14.8
*ermC*	Erythromycin/clindamycin resistance	28	9.0
*linA*	Lincosamide nucleotidyltransferase	31	10.0
*msrA*	energy-dependent efflux of erythromycin	18	5.8
*vatB*	Acetyl-transferase inactivating streptogramin A	2	0.6
*vgaA*	ATP binding protein, streptogramin A resistance	2	0.6
*aacA-aphD*	Bifunctional enzyme gentamicin resistance	42	13.5
*aadD*	Aminoglycoside adenyl-transferase, tobramycin resistance	43	13.8
*aphA3*	3′5′-aminoglycoside phosphotransferase, neo-/kanamycin resistance	56	18.0
*sat*	Streptothricin acetyltransferase	36	11.6
*dfrA*	Dihydrofolate reductase type 1	12	3.9
*far1*	Fusidic acid resistance (plasmid-borne)	3	1.0
*Q6GD50 (fusC)*	SCC-associated fusidic acid resistance	44	14.1
*mupA*	Mupirocin resistance protein	28	9.0
*tetK*	Tetracycline resistance	9	2.9
*tetM*	Tetracycline resistance	1	0.3
*cat*	Chloramphenicol acetyltransferase	3	1.0
*fexA*	Chloramphenicol/florfenicol exporter	121	38.9
*fosB*	Metallothiol transferase	53	17.0
*fosB-plasmid*	Metallothiol transferase	0	0.0
*qacA*; *qacC*	Quaternary ammonium compound resistance protein A/B	4	1.3
*tetEfflux*	Transport-/efflux protein	90	28.9
*vanA*; *vanB*	Vancomycin resistance genes	0	0.0

**Table 4:** Detection of virulence genes.

Virulence Genes		# Positive (N = 311)	% Positive
*tst1*	Toxic shock syndrome toxin 1	38	12.2
*lukF-PV*; *lukS-PV*	Panton Valentine leukocidin F/S component	120	38.5
*sak*	Staphylokinase	146	46.9
*chp*	Chemotaxis-inhibiting protein	225	72.3
*scn*	Staphylococcal complement inhibitor	79	25.4
*etA*	Exfoliative toxin serotype A	0	0
*etB*	Exfoliative toxin serotype B	3	0.9
*etD*	Exfoliative toxin D	6	1.9
*edinA*	Epidermal cell differentiation inhibitor A	21	6.8
*edinB*	Epidermal cell differentiation inhibitor B	4	1.3
*edinC*	Epidermal cell differentiation inhibitor C	179	57.6
ACME	Arginine catabolic mobile element locus	17	5.5
*sasG*	*Staphylococcus aureus* surface protein G	128	41.2

## Data Availability

All relevant data are provided in the main text of this manuscript.

## Supplementary Materials

The following supporting information can be downloaded at: https://www.mdpi.com/article/10.3390/antibiotics14010024/s1, Table S1: SCCmec types identified.
