# Supplementary material for: Methicillin-Resistant Staphylococcus aureus: The Shifting Landscape in the United Arab Emirates
Source: Antibiotics (Basel). 2025 Jan 2;14(1):24. doi: 10.3390/antibiotics14010024 (PMC11759823; doi:10.3390/antibiotics14010024)
Supplement: Supplementary file 1 [file antibiotics-14-00024-s001.zip › antibiotics-3228713-supplementary.pdf]

Table S1. SCC*mec* types identified

**SCC*mec* Type**	**Number of isolates**	**Percentage**
**II**	2	0.7
**III**	1	0.3
**IV**	8	2.6
**V/VT**	153	49.3
**VI**	129	41.6
**Others ***	17	5.5

* PseudoSCC*mec* [classC+*fus*+*ccrAB1*] (n=1), pseudo SCC*mec* IV (n=1), PseudoSCC*mec* [class B+*fus*+*ccrAB1*] (n=2), Only clonal complex (n=13)
